# Factors associated with prolonged overall survival in patients with postmenopausal estrogen receptor-positive advanced breast cancer using real-world data: a follow-up analysis of the JBCRG-C06 Safari study

**DOI:** 10.1007/s12282-019-01029-3

**Published:** 2019-12-06

**Authors:** Hidetoshi Kawaguchi, Norikazu Masuda, Takahiro Nakayama, Kenjiro Aogi, Keisei Anan, Yoshinori Ito, Shoichiro Ohtani, Nobuaki Sato, Shigehira Saji, Toshimi Takano, Eriko Tokunaga, Seigo Nakamura, Yoshie Hasegawa, Masaya Hattori, Tomomi Fujisawa, Satoshi Morita, Miki Yamaguchi, Hiroko Yamashita, Toshinari Yamashita, Yutaka Yamamoto, Daisuke Yotsumoto, Masakazu Toi, Shinji Ohno

**Affiliations:** 1 Department of Breast Surgery, Matsuyama Red Cross Hospital, 1 Bunkyo-cho, Matsuyama, 790-8524 Japan; 2 Department of Surgery, Breast Oncology, NHO Osaka National Hospital, Osaka, 540-0006 Japan; 3 Department of Breast and Endocrine Surgery, Osaka International Cancer Institute, Osaka, 541-8567 Japan; 4 Department of Breast Oncology, NHO Shikoku Cancer Center, Matsuyama, 791-0280 Japan; 5 Department of Surgery, Kitakyushu Municipal Medical Center, Kitakyushu, 802-0077 Japan; 6 Department of Breast Medical Oncology, The Cancer Institute Hospital of JFCR, Tokyo, 135-8550 Japan; 7 Department of Breast Surgery, Hiroshima City Hiroshima Citizens Hospital, Hiroshima, 730-8518 Japan; 8 Department of Breast Oncology, Niigata Cancer Center Hospital, Niigata, 951-8566 Japan; 9 Department of Medical Oncology, Fukushima Medical University, Fukushima, 960-1295 Japan; 10 Department of Medical Oncology, Toranomon Hospital, Tokyo, 105-8470 Japan; 11 Department of Breast Oncology, Kyushu Cancer Center, Fukuoka, 811-1395 Japan; 12 Department of Surgery, Division of Breast Surgical Oncology, Showa University School of Medicine, Tokyo, 142-8666 Japan; 13 Department of Breast Surgery, Hirosaki Municipal Hospital, Hirosaki, 036-8004 Japan; 14 Department of Breast Oncology, Aichi Cancer Center Hospital, Nagoya, 464-8681 Japan; 15 Department of Breast Oncology, Gunma Prefectural Cancer Center, Ohta, 373-8550 Japan; 16 Department of Biomedical Statistics and Bioinformatics, Graduate School of Medicine, Kyoto University, Kyoto, 606-8507 Japan; 17 Department of Breast Surgery, JCHO Kurume General Hospital, Kurume, 830-0013 Japan; 18 Department of Breast Surgery, Hokkaido University Hospital, Sapporo, 060-8648 Japan; 19 Department of Breast and Endocrine Surgery, Kanagawa Cancer Center, Yokohama, 241-8515 Japan; 20 Department of Breast and Endocrine Surgery, Kumamoto University Graduate School of Medical Sciences, Kumamoto, 860-8556 Japan; 21 Department of Breast Surgical Oncology, Hakuaikai Medical Corporation Sagara Hospital, Kagoshima, 892-0833 Japan; 22 Department of Breast Surgery, Graduate School of Medicine, Kyoto University, Kyoto, 606-8507 Japan; 23 Breast Oncology Center, The Cancer Institute Hospital of JFCR, Tokyo, 135-8550 Japan

**Keywords:** Breast cancer, Antineoplastic agents, Post-menopause, Hormones, Overall survival

## Abstract

**Background:**

Assessing survival risk is important for discussing treatment options with estrogen receptor-positive (ER+) advanced breast cancer (ABC) patients. However, there are few reports from large-scale databases on the survival risk factors in ER+ ABC. The Safari study (UMIN000015168) was a retrospective, multicenter cohort study involving 1072 Japanese patients receiving fulvestrant 500 mg mostly as a second- or later-line endocrine therapy for ER+ ABC. The follow-up data after the Safari study were examined, focusing on any relationship between clinicopathological factors and overall survival (OS) in ER+ ABC patients.

**Methods:**

OS in patients with ER+ ABC was analyzed by univariate and multivariate analyses with a Cox proportional hazards model in this study.

**Results:**

A total of 1031 cases were evaluable for OS analysis. Multivariate analysis showed that younger age (< 60 years), longer time from ABC diagnosis to fulvestrant use (≥ 3 years), no prior palliative chemotherapy before fulvestrant use, and progesterone receptor (PgR) negativity (PgR−) were significantly correlated with prolonged OS (median 7.0 years). For cases with histological or nuclear grade data, lower histological or nuclear grades were also correlated with longer OS. In recurrent metastatic cases, long disease-free interval (DFI) was not correlated with longer OS.

**Conclusions:**

In ER+ ABC patients whose treatment history included fulvestrant, younger age, longer time from ABC diagnosis to fulvestrant use, no prior palliative chemotherapy use, PgR−, and lower histological or nuclear grade correlated positively with prolonged OS.

**Electronic supplementary material:**

The online version of this article (10.1007/s12282-019-01029-3) contains supplementary material, which is available to authorized users.

## Introduction

Most breast cancer patients who are diagnosed at an early stage have a good clinical outcome [1]. However, nearly 30% of patients newly diagnosed with early-stage breast cancer will later develop recurrent metastatic cancer [2], for whom the 5-year survival rate is approximately 20% [3]. In advanced breast cancer (ABC) (including locally advanced and metastatic breast cancer [4]) patients, the disease currently remains incurable [5]. In ABC, estrogen receptor-positive (ER+) is the most common subtype [6].

Recently, several drugs, such as selective estrogen receptor modulators (SERMs), selective estrogen receptor downregulators (SERD), aromatase inhibitors (AIs), cyclin dependent kinase (CDK) 4/6 inhibitors, and mTOR inhibitor have been used to treat ER+ ABC. Other chemotherapy regimens as well as bevacizumab are also effective, especially for high-risk cases [7] or cases suspected to be endocrine therapy resistant. Selecting drugs to maximize overall survival (OS) and quality of life for ER+ ABC is obviously desirable. Unfortunately, the tumor burden and tumor biology differ between initial diagnosis and recurrence, and patients may have become endocrine therapy resistant because they continued endocrine therapy as adjuvant treatment [8, 9]. However, there are few reports from large-scale data sets on the prognostic factors for ABC [10–12], especially focusing on ER+ ABC [6, 13, 14].

We constructed a database of more than 1000 ER+ breast cancer cases in the Safari study. The Safari study examined the association between clinicopathological factors and time to treatment failure (TTF) of fulvestrant in Japanese ABC patients who received fulvestrant [15, 16]. Fulvestrant is a selective estrogen receptor degrader that is administered to patients with ER+ ABC [17, 18]. We continued to collect prognostic data after the main analysis of the Safari study was completed.

The objective of this OS analysis was to examine the data from the Safari study with a focus on the potential effect that patient- or disease-related factors [19] may have on OS.

## Materials and methods

### OS analysis design

The study design and patient cohort for the Safari study (UMIN000015168) have been published previously [15, 16]. In brief, Safari study was a retrospective, multicenter cohort study that examined the association between clinicopathological factors and TTF of fulvestrant in Japanese ABC (JBCRG-C06 Safari) [15, 16]. The study was conducted in accordance with the Helsinki Declaration, “Guidelines for Clinical Evaluation Methods of Anti-Cancer Drugs” and “Ethical Guidelines for Epidemiology Research (revised on December 1, 2008)”. The protocol was approved at each institute. Patients were followed according to the guidelines of the Japan Breast Cancer Society and the National Comprehensive Cancer Network (NCCN) Guidelines for Invasive Breast Cancer (Version 4.2018) [20], which is the standard treatment in Japan. This study was registered as UMIN000015168.

Patients starting fulvestrant treatment between 25 November 2011 (fulvestrant approval date in Japan) and 31 December 2014 were registered. The data cut-off for primary analysis was 27 September 2015 and for OS analysis was 30 April 2018.

We analyzed OS using the same factors of TTF as previously reported [15, 16] because we wanted to compare the results of this study with those of our previous report regarding TTF. Factors investigated were patient age (≥ 60 years vs. < 60 years), fulvestrant treatment line (≥ 4th vs. 3rd vs. 1st and 2nd), period from ABC diagnosis to fulvestrant use (≥ 3 years vs. < 3 years), histological or nuclear grade (2 vs. 1, 3 vs. 1), visceral metastasis (yes vs. no), progesterone receptor (PgR) expression (positive vs. negative), human epidermal growth factor receptor 2 (HER2) expression (positive vs. negative), disease-free interval (DFI) (≥ 5 vs. < 5 years), and prior palliative chemotherapy use (yes vs. no). “Prior palliative chemotherapy use” means any chemotherapy received after the diagnosis of ABC to the start of fulvestrant, and excludes any neoadjuvant or adjuvant chemotherapy.

In this analysis, all cut-off other than age and DFI were matched to the main analysis of TTF. The median age of 65 years was the cut-off in the TTF analysis, and the median age of 60 years was the cut-off in the OS analysis. The reason why the median age differed between the TTF analysis and the OS analysis is because the starting point of each analysis was different. The starting point for the TTF analysis was the start date of the fulvestrant treatment, while the starting point of the OS analysis was the start of treatment for advanced cancer, and the median age at that time was 5 years younger than the start of treatment for fulvestrant. Regarding the DFI cut-off, in our previous report we used "DFI (≥ 2 vs. < 2 years)". We used a cut-off of 2 years, as explained in the ESO–ESMO guidelines about intrinsic or primary endocrine resistance for early breast cancer, if defined as recurrence within the first 2 years of adjuvant endocrine therapy [4]. Nonetheless, in this analysis, we wanted to examine factors related to OS rather than endocrine therapy sensitivity, so the cut-off was set at a median of 5 years instead of 2 years.

### Statistical methods

OS was defined as the duration from the date of initial treatment of recurrent breast cancer to death. Kaplan–Meier curves were also used for analysis to investigate OS. Subjects whose deaths were not documented by the time of data cut-off were censored when survival was last documented. The Cox hazards model was used to evaluate the relationship between each of the clinical factors and OS. We also performed multivariate analysis on OS using factors that had a statistical difference (*P* < 0.1) in univariate analysis. Hazard ratios (HRs) with 95% confidence intervals (CIs) and *P* values are described. All tests were two-sided and *P* < 0.05 was considered statistically significant.

## Results

### Patient demographics

**Fig. 1:**
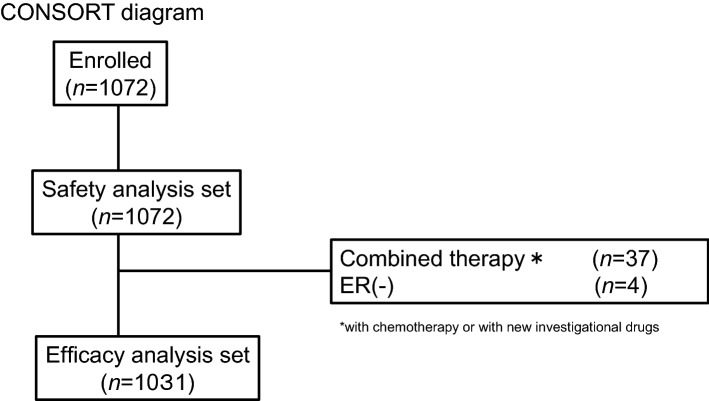
Patient flow diagram. *ER* estrogen receptor, *HER2* human epidermal growth factor receptor 2

A total of 1072 cases from 16 centers were enrolled in the Safari study. A CONSORT diagram is shown in Fig. 1. Thirty-seven cases were not eligible because they combined fulvestrant with other therapies (such as endocrine therapy, and/or chemotherapy, and/or target therapy). However, HER2-combined cases were allowed. Four cases were also excluded from the analysis because they were estrogen receptor-negative (ER−). Finally, 1031 (96.2%) patients with ER+ ABC were eligible for the OS analysis. After the main analysis of TTF, we collected follow-up data for treatment and OS. When we collected these data, we queried PgR and HER2 status because there were some unknown or unclear cases. For the receptor status determination, data that could be determined before the administration of fulvestrant were used in the analysis of TTF, but in this study, data that could be determined when ABC was diagnosed were used. As a result, PgR and HER2 status were changed as follows: PgR positivity went from 805 to 765 cases, negativity from 187 to 198 cases, and unknown from 39 to 68 cases, while HER2 positivity went from 117 to 94 cases, negativity from 819 to 884 cases, and unknown from 95 to 53 cases (Supplementary Table 1).

Table 1 shows the baseline characteristics of OS data sets. The median age was 60.0 years, visceral metastasis was found in 408 (57.5%) cases, and central nervous metastasis was observed in eight (1.4%) cases. Most cases were ER+ PgR + , and fulvestrant was used most often for late-line treatment (4th line or later).

**Table 1:** Summary of patient characteristics

	Efficacy analysis sets	De novo metastatic or locally advanced	Recurrent metastatic
Characteristic	*n* = 1031	*n* = 207	*n* = 824
Age (ABC diagnosis), years			
Median	60.0	60.0	60.0
Range	29–91	35–90	29–91
Age group (ABC diagnosis), (*n* [%])			
< 60	486 (47.1)	97 (46.9)	389 (47.2)
≥ 60	545 (52.9)	110 (53.1)	435 (52.8)
Fulvestrant treatment lines			
1st	21 (2.0)	3 (1.4)	18 (2.2)
2nd	232 (22.5)	50 (24.2)	182 (22.1)
3rd	276 (26.8)	59 (28.5)	217 (26.3)
4th or more	502 (48.7)	95 (45.9)	407 (49.4)
ABC diagnosis to fulvestrant use, years			
Median	3.4	3.2	3.4
Range	0–26.9^a^	0–18.2	0–26.9^b^
ABC diagnosis to fulvestrant use, group, years			
< 3	473 (45.9)	96 (46.4)	377 (45.8)
≥ 3	557 (54.0)	111 (53.6)	446 (54.1)
Missing	1 (0.1)	0	1 (0.1)
DFI, years			
Median	NA	NA	5.5
Range	NA	NA	0–31.8^b^
DFI, group, years			
< 5	NA	NA	380 (46.1)
≥ 5	NA	NA	443 (53.8)
Missing	NA	NA	1 (0.1)
Visceral metastasis			
No	588 (57.0)	106 (51.2)	482 (58.5)
Yes	443 (43.0)	101 (48.8)	342 (41.5)
Central nerve metastasis			
No	1017 (98.6)	203 (98.1)	814 (98.8)
Yes	14 (1.4)	4 (1.9)	10 (1.2)
Histological type			
IDC	863 (83.7)	173 (83.6)	690 (83.7)
ILC	48 (4.7)	7 (3.4)	41 (5.0)
Other	120 (11.6)	27 (13.0)	93 (11.3)
Histological or nuclear grade			
1	314 (30.5)	48 (23.2)	266 (32.3)
2	240 (23.3)	54 (26.1)	186 (22.6)
3	125 (12.1)	28 (13.5)	97 (11.8)
NA	352 (34.1)	77 (37.2)	275 (33.4)
Hormonal receptor			
ER(+) PgR(−)	198 (19.2)	36 (17.4)	162 (19.7)
ER(+) PgR(+)	765 (74.2)	159 (76.8)	606 (73.5)
ER( +) PgR(NA)	68 (6.6)	12 (5.8)	56 (6.8)
HER2			
Negative	884 (85.7)	174 (84.1)	710 (86.2)
Positive	94 (9.1)	21 (10.1)	73 (8.9)
Missing	53 (5.1)	12 (5.8)	41 (5.0)
Prior palliative chemotherapy use			
No	548 (53.2)	95 (45.9)	453 (55.0)
Yes	483 (46.8)	112 (54.1)	371 (45.0)

Results are *n* (%) unless otherwise noted

*ABC* advanced breast cancer, *DFI* disease-free interval, *ER* estrogen receptor, *HER2* human epidermal growth factor receptor 2, *IDC* invasive ductal carcinoma, *ILC* invasive lobular carcinoma, *PgR* progesterone receptor

^a^*n* = 1030

^b^*n* = 823

## Analysis outcomes

### Analysis of all cases

**Fig. 2:**
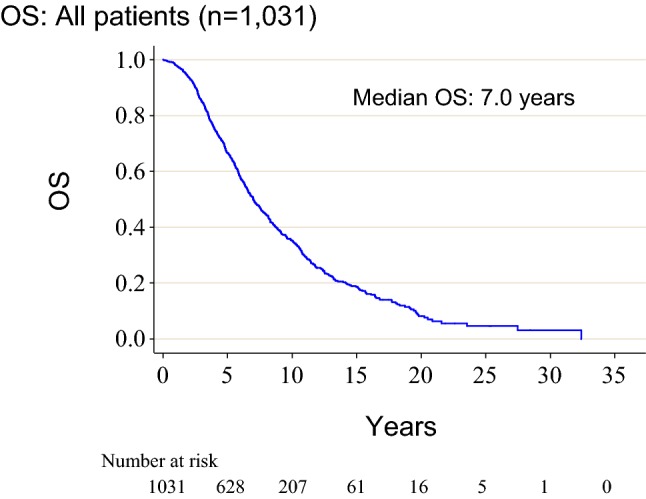
Kaplan–Meier estimates for OS in all cases. *OS* overall survival

Median OS was 7.0 years (95% CI 6.6–7.5 years) (Fig. 2). By univariate analysis (Table 2), younger age (< 60 years), earlier treatment line of fulvestrant, longer time from ABC diagnosis to fulvestrant use (≥ 3 years), no prior palliative chemotherapy use, lower histological or nuclear grade, and PgR negativity (PgR−) were associated with longer OS (*P* < 0.1). In the treatment line of fulvestrant, visceral metastases and HER2 status had no effect on OS (Table 2).

**Table 2:** Univariate and multivariate Cox proportional hazards regression models for OS: all patients (*n* = 1031)

Characteristic	Univariate analysis			Multivariate analysis^a^		
	HR	95% CI	*p* value	HR	95% CI	*P* value
Age (ABC diagnosis) (≥ 60 years vs. < 60)	1.59	1.36–1.85	< 0.001	1.35	1.15–1.58	< 0.001
Treatment line of fulvestrant (4th or more vs. 3rd vs. 1st and 2nd)	0.68	0.61–0.75	< 0.001	1.00	0.90–1.12	0.965
ABC diagnosis to fulvestrant use (≥ 3 years vs. < 3)	0.16	0.13–0.19	< 0.001	0.14	0.12–0.18	< 0.001
Prior palliative chemotherapy (yes vs. no)	0.78	0.67–0.90	0.001	1.44	1.21–1.71	< 0.001
Histological or nuclear grade						
(2 vs. 1)	1.74	1.45–2.08	< 0.001			
(3 vs. 1)	2.30	1.86–2.85	< 0.001			
Visceral metastasis (yes vs. no)	0.97	0.83–1.13	0.685			
PgR (positive vs. negative)	1.17	0.98–1.39	0.089	1.29	1.06–1.59	0.013
HER2 (positive vs. negative)	0.84	0.65–1.09	0.200			

*OS* overall survival, *HR* hazard ratio, *CI* confidence interval, *ABC* advanced breast cancer, *PgR* progesterone receptor HER2, human epidermal growth factor receptor 2

^a^*n* = 1030 in the multivariate analysis (one patient was excluded because date of recurrence was unknown. Histological/nuclear grade data were not included in this analysis because one-third of the data were missing

By multivariate analysis, younger age (< 60 years; *P* < 0.001), longer time from ABC diagnosis to fulvestrant use (≥ 3 years; *P* < 0.001), no prior palliative chemotherapy use (*P* < 0.001), and PgR– (*P* = 0.013) were correlated with prolonged OS (Table 2). Histological or nuclear grade were not included in this multivariate analysis because data were missing in one-third of cases.

### Analysis of all cases with available histological or nuclear grade data

A subgroup analysis was performed on 679 cases that had histological or nuclear grade data. The patients used in this analysis decreased from 691 cases of TTF to 679 cases. The reason was that the TTF analysis was targeted to those whose histological or nuclear grade could be determined before administration of fulvestrant, but in this study, it was limited to cases that could be determined at the time of diagnosis of ABC. In other words, grade data includes data from the first diagnosis of breast cancer and metastatic site data at the time of recurrence. However, data obtained during palliative treatment from the diagnosis of ABC has not been included, even from a biopsy of a local recurrence site or a metastatic recurrence site. Multivariate analysis revealed that lower histological or nuclear grade was also correlated with significantly longer OS (2 vs. 1; *P* = 0.021) (3 vs. 1; *P* < 0.001) (Table 3).

**Table 3:** Multivariate Cox proportional hazards regression models for OS using a dataset of all cases had grade information (*n* = 679)

Characteristic	HR	95% CI	*p* value
Age (ABC diagnosis), (≥ 60 vs. < 60 years)	1.16	0.96–1.40	0.130
Treatment line of fulvestrant (4th or more vs. 3rd vs. 1st and 2nd)	1.02	0.90–1.16	0.754
ABC diagnosis to fulvestrant use (≥ 3 vs. < 3 years)	0.18	0.14–0.24	< 0.001
Prior palliative chemotherapy (yes vs. no)	1.37	1.12–1.67	0.002
PgR (positive vs. negative)	1.20	0.95–1.53	0.131
Histological or nuclear grade			
(2 vs. 1)	1.28	1.04–1.59	0.021
(3 vs. 1)	1.78	1.39–2.28	< 0.001

*OS* overall survival, *HR* hazard ratio, *CI* confidence interval, *ABC* advanced breast cancer, PgR, progesterone receptor

### Analysis of recurrent metastatic cases

We analyzed the correlation between DFI and OS in recurrent metastatic cases (*n* = 824). By univariate analysis (Table 4), younger age (< 60 years), earlier treatment line of fulvestrant, longer time from ABC diagnosis to fulvestrant use (≥ 3 years), no prior palliative chemotherapy, and lower histological or nuclear grade were associated with longer OS (*P* < 0.1). By multivariate analysis, younger age (< 60 years; *P* < 0.0001), longer time from ABC diagnosis to fulvestrant use (≥ 3 years; *P* < 0.001), and no prior palliative chemotherapy (*P* < 0.001) were associated with significantly longer OS (Table 4). DFI was not a factor related to OS. Histological or nuclear grade were not included in this multivariate analysis because data were missing in one-third of cases.

**Table 4:** Univariate and multivariate Cox proportional hazards regression models for OS using a dataset of recurrent metastatic cases (*n* = 824)

Characteristic	Univariate analysis			Multivariate analysis^a^		
	HR	95% CI	*p* value	HR	95% CI	*p* value
Age (ABC diagnosis), (≥ 60 vs. < 60 years)	1.66	1.40–1.98	< 0.001	1.42	1.19–1.70	< 0.001
Treatment line of fulvestrant (4th or more vs. 3rd vs. 1st and 2nd)	0.70	0.62–0.78	< 0.001	1.00	0.89–1.13	0.992
ABC diagnosis to fulvestrant use (≥ 3 vs. < 3 years)	0.16	0.13–0.19	< 0.001	0.14	0.11–0.18	< 0.001
Prior palliative chemotherapy (yes vs. no)	0.78	0.66–0.93	0.004	1.45	1.20–1.76	< 0.001
Histological or nuclear grade						
(2 vs. 1)	1.73	1.41–2.12	< 0.001			
(3 vs. 1)	2.58	2.03–3.27	< 0.001			
Visceral metastasis (yes vs. no)	0.99	0.83–1.17	0.898			
PgR (positive vs. negative)	1.14	0.94–1.38	0.200			
HER2 (positive vs. negative)	0.87	0.65–1.17	0.360			
DFI (≥ 5 vs. < 5 years)	1.00	0.84–1.18	0.950			

*OS* overall survival, *HR* hazard ratio, *CI* confidence interval, *ABC* advanced breast cancer, *PgR* progesterone receptor HER2, human epidermal growth factor receptor 2, *DFI* disease-free interval

^a^*n* = 823 in the multivariate analysis (one patient was excluded because the date of recurrence was unknown. Histological/nuclear grade data were not included in this analysis because one-third of the data were missing

### Analysis of recurrent metastatic cases with available histological or nuclear grade data

Multivariate analysis for OS in recurrent metastatic cases for which histological or nuclear grades were known showed that lower histological or nuclear grade also correlated with significantly longer OS (3 vs. 1; *P* < 0.001) (Table 5).

**Table 5:** Multivariate Cox proportional hazards regression models for TTF using a dataset of recurrent metastatic cases that had grade information (*n* = 549)

Characteristic	HR	95% CI	*p* value
Age (ABC diagnosis), (≥ 60 vs. < 60 years)	1.27	1.03–1.57	0.026
Treatment line of fulvestrant (4th or more vs. 3rd vs. 1st and 2nd)	1.00	0.87–1.14	0.964
ABC diagnosis to fulvestrant use (≥ 3 vs. < 3 years)	0.18	0.13–0.24	< 0.001
Prior palliative chemotherapy use (yes vs. no)	1.41	1.12–1.77	0.003
Histological or nuclear grade			
(2 vs. 1)	1.21	0.95–1.53	0.122
(3 vs. 1)	1.95	1.48–2.57	< 0.001

*OS* overall survival, *HR* hazard ratio, *CI* confidence interval, *ABC* advanced breast cancer, *PgR* progesterone receptor HER2

^a^Histological or nuclear grade data are included (*n* = 558)

## Discussion

This study was an OS analysis of the large-scale, retrospective Safari study, a cohort study that analyzed the clinical outcomes of fulvestrant-treated ER+ ABC patients in Japan [15, 16]. The median OS in ER+ patients was 7.0 years in our study, which was longer than the OS from similar cohort studies (3–5 years) [21–23]. One reason may be that the database of the Safari study included all cases of fulvestrant administration, including good-prognosis cases receiving fulvestrant after long-term endocrine therapy. Another reason may be the so-called lead-time bias, because physicians usually test tumor markers for follow-up of breast cancer patients after surgery in Japan [24]. “Lead time” is the length of time between the detection of a disease and its usual clinical presentation and diagnosis. By screening, the intention is to diagnose a disease earlier than it would be without screening. Without screening, the disease may be discovered later, when symptoms appear [25]. Another possibility is that Japan has a universal health insurance system, and anyone can receive insurance coverage [26, 27]. Therefore, there are few cases of patients discontinuing treatment because of cost, and it can be considered that continuing treatment longer than overseas cases is another reason for longer OS [26, 27].

A critical purpose of this subgroup analysis was to examine the data from the Safari study with a focus on the potential effect that patient- or disease-related factors may have on OS for ER+ ABC. Identifying predictive factors of poor prognosis for ABC is very important for decision-making in patient treatment. In this study, multivariate analysis showed that younger age [22, 28] and low histological or nuclear grade [29] correlated with longer OS, as previously reported. Age is a patient-related factor, while histological or nuclear grade is disease-related [19]. Moreover, no prior palliative chemotherapy was also associated with significantly longer OS. Physicians are considered to have the tendency to administer chemotherapy at an earlier line for cases judged to be endocrine resistant and that have a poor prognosis. Our study suggested that in clinical practice the former is correct.

One interesting finding from this study is the association between a longer period from ABC diagnosis to fulvestrant use and longer OS. This finding may indicate that patients who respond well to other prior therapies will also respond to fulvestrant, implying that a durable response to the previous drug correlates with a durable response to fulvestrant. The above four factors other than age, lower histological or nuclear grade, longer time from ABC diagnosis to fulvestrant use, and no prior palliative chemotherapy, were also factors with longer TTF of fulvestrant [15, 16]. Because the fulvestrant treatment line varied in this study, the fulvestrant TTF length and OS length of ER+ ABC patients cannot simply be compared, but the fulvestrant TTF length may be a surrogate marker for subsequent OS.

Previous studies have also demonstrated that longer DFI [10–12, 22] and no visceral metastasis [10–13, 22] were positively correlated with longer OS in ABC. However, these factors were not associated with OS in our multivariate analysis. In this cohort, the cases with common poor prognostic factors, including short DFI and visceral metastases, might have had endocrine therapy discontinued prior to fulvestrant use, which was mostly used as a second- or later-line endocrine therapy, and shifted to chemotherapy without being treated with fulvestrant. Consequently, these patients were not included in the analyzed cohort, which may be the reason why, in this cohort, multivariate analysis did not extract these prognostic factors. Regarding the DFI cut-off, described in our previous report about TTF, we used "DFI (≥ 2 vs. < 2 years)". We used a cut-off of 2 years, as explained in the ESO–ESMO guidelines about intrinsic or primary endocrine resistance for early breast cancer, if defined as recurrence within the first 2 years of adjuvant endocrine therapy [4]. Nonetheless, in this analysis, we wanted to examine factors related to OS rather than endocrine therapy sensitivity, so the cut-off was set at a median of 5 years instead of 2 years. In fact, we also performed an analysis with a cut-off of 2 years, but the multivariate analysis did not show any correlation between DFI and OS (data not shown).

One confusing result of our study is that PgR− correlated with longer OS by multivariate analysis, which is the opposite to previous reports [10, 11]. This result is difficult to explain logically, however, probably due to the selection bias of this study or confounding factors not defined in our analysis.

In recent years, molecular targeting drugs such as CDK 4/6 inhibitors and mTOR inhibitor have been administered in ER+ HER2 − ABC in combination with endocrine therapy, and endocrine therapy plus molecular targeted therapy is administered as an early-line treatment for ER+ HER2− ABC. At present, it is not possible to obtain data on the sequential administration of hormonal monotherapy as in the Safari study, so the significance of creating this database is profound. Even in the era of molecular targeting therapy combinations, continuation of molecular targeting drugs after tumor progression, so-called “beyond progression”, is not recommended [30], and analysis using data of sequential administration of hormonal therapy that is the basis of ER+ HER2− breast cancer treatment is important. In the future, we plan to conduct research to analyze the effect of treatment order on OS by dynamic treatment regimen analysis [31] using the database from the Safari study.

As with the original Safari study, the limitations of our analysis include its retrospective design and the absence of a comparative treatment group. Moreover, there is a high bias due to selection of the fulvestrant-treated cohort, which probably does not reflect the ER+ ABC population. In addition, our results are considered to reflect real clinical practice. We initiated this study to examine the factors in the main analysis that affect fulvestrant TTF. Therefore, the OS analysis also included the treatment line of fulvestrant, the period from ABC diagnosis to fulvestrant use, and palliative chemotherapy prior to fulvestrant administration. We did not consider other endocrine therapies, and thus this is also a limitation of this study.

In conclusion, in ER+ ABC patients who received fulvestrant, younger age, longer period from recurrent breast cancer diagnosis to fulvestrant use, and low histological or nuclear grade, were associated with longer OS. These factors may be the key to predicting poor prognosis in patients with ER+ ABC whose treatment history includes fulvestrant.

## Electronic supplementary material

Below is the link to the electronic supplementary material.

Supplementary file1 (DOCX 16 kb)
