# Supplementary material for: Factors associated with prolonged overall survival in patients with postmenopausal estrogen receptor-positive advanced breast cancer using real-world data: a follow-up analysis of the JBCRG-C06 Safari study
Source: Breast Cancer. 2019 Dec 6;27(3):389–98. doi: 10.1007/s12282-019-01029-3 (PMC7196081; doi:10.1007/s12282-019-01029-3)
Supplement: Supplementary file 1 — Supplementary file1 (DOCX 16 kb) [file 12282_2019_1029_MOESM1_ESM.docx]

**Supplementary Table 1.** Receptor status of TTF analysis and OS analysis

ER PgR HER2

*n*=1031 *n*=1031 *n*=1031

TTF OS TTF OS TTF OS

Negative 0 (0) 0 (0) 187 (18.1) 198 (19.2) 819 (79.4) 884 (85.7)

Positive 1031 (100) 1031 (100) 805 (78.1) 765 (74.2) 117 (11.3) 94 (9.1)

Unknown 0 (0) 0 (0) 39 (3.8) 68 (6.6) 95 (9.2) 53 (5.1)

Results are n (%) unless otherwise noted.

TTF, time to treatment failure; OS, overall survival; ER, estrogen receptor; PgR, progesterone receptor; HER2, human epidermal growth factor receptor 2.
